# Burden of hepatocellular carcinoma among hispanics in South Texas: a systematic review

**DOI:** 10.1186/s40364-017-0096-5

**Published:** 2017-04-21

**Authors:** John Ha, Aysha Chaudhri, Abhishek Avirineni, Jen-Jung Pan

**Affiliations:** 1 Department of Internal Medicine, The University of Texas Health Science Center at Houston, 6431 Fanin St, MSB 1.150, Houston, TX 77030 USA; 2 Division of Gastroenterology, Hepatology and Nutrition, Department of Internal Medicine, The University of Texas Health Science Center at Houston, 6431 Fanin St, MSB 4.234, Houston, TX 77030 USA

**Keywords:** Hepatocellular carcinoma, Race disparities, Ethnic disparities, Non-alcoholic fatty liver disease, Metabolic syndrome, Healthcare access

## Abstract

**Background:**

Hepatocellular carcinoma (HCC) is one of the fastest rising causes of cancer-related mortality in the United States (U.S.). Despite improved HCC screening and surveillance guidelines, significant race/ethnicity-specific disparities in hepatocellular carcinoma remain, disproportionately affecting at risk racial minorities in the U.S. The current review aims to provide an updated analysis on race/ethnicity-specific disparities in HCC epidemiology with a focus on predisposing risk factors.

**Conclusion:**

Among different race/ethnicities in the U.S., Hispanics experienced the greatest burden of HCC, particularly those residing in South Texas. It is important to understand that the underlying etiologies for these disparities are complex and multi-factorial. Some of these risk factors for developing chronic liver disease include non-alcoholic fatty liver disease/non-alcoholic steatohepatitis and alcohol use. In addition, population genetics, acculturation of ethnic minorities, and access to healthcare may be further contributing to the observed disparities in HCC. By increasing awareness, improved modalities for screening and surveillance for HCC are important in guiding future research for targeted preventive and therapeutic interventions.

## Background

Hepatocellular Carcinoma (HCC) is the fifth leading cause of cancer-related death in the United States (U.S.) among men and ninth in women [1]. In the U.S., the incidence of HCC has more than doubled since the 1980’s, becoming one of the fastest rising causes of cancer-related mortality, which may be attributed to the rising trend of chronic Hepatitis C Virus infection (HCV) and incidence of non-alcoholic fatty liver disease (NAFLD) [2]. Despite improvements in screening and surveillance efforts for HCC, the overall 5-year survival for HCC remains dismal. According to the Surveillance, Epidemiology, and End Results (SEER) registry, the overall 5-year survival for localized HCC is less than 31%, with more advanced HCC having significantly worse outcomes [3].

Among different race/ethnic populations in the U.S., significant disparities in HCC have been observed in recent years, where Hispanics experienced the greatest increase in HCC incidence by approximately 36% [4]. Hispanics accounted for nearly 50% of the U.S. population growth from 2000 to 2010 and are projected to reach 30% of the U.S. population within the next three decades [5]. It is conceivable for the incidence of HCC in the U.S. to increase proportionally with the rising Hispanic population. Although Texas accounts for the second greatest distribution of Hispanics by state, the incidence of HCC among Hispanics in Texas, particularly South Texas, was significantly higher than elsewhere in the U.S. [6]. This trend may be caused by the prevalence of unique regional risk factors, such as metabolic syndrome, NAFLD, population genetics, and different environmental exposures. The current review aims to provide an updated epidemiological analysis of the Hispanic cohort in South Texas and future implications of HCC in the region.

## Epidemiology of HCC

HCC remains a leading cause of cancer-related morbidity and mortality in the U.S. There are over 20,000 new cases of HCC in the U.S. per year, where the incidence has tripled over the last three decades [7, 8]. Through analysis of the 2003–2011 SEER database, Ha et al. demonstrated the age-adjusted HCC incidence to be 9.4/100,000 (95% confidence interval (CI) 9.3–9.5). When evaluating HCC incidence between race/ethnicities, Asians has the highest overall incidence of HCC (18.6/100,000; 95% CI 18.2–19.0), followed by African Americans (15.7/100,000; 95% CI 15.4–16.1), Hispanics (11.8/100,000; 95% CI 11.6–12.2), and Non-Hispanic whites (7.0/100,000; 95% CI 6.9–7.1). During the same period, Hispanics were seen to have the greatest increase in HCC incidence (+35.8%) with an annual percent change of 4% [4]. When observing the overall trend in the incidence of HCC in the U.S., recent studies suggest HCC incidence to have stabilized; however, this trend may not be reflected among Hispanics residing in Texas, a region not included in the national SEER registry [4, 9, 10].

Compared to other 49 states in the U.S., Texas reported the highest age-adjusted incidence of HCC [11]. Hispanics residing in Texas make up approximately 20% of the overall U.S. Hispanic population, where 2,500 new cases of HCC are seen annually within this race/ethnic cohort [6, 12]. Approximately 70% of the population in South Texas is Hispanic, where the region accounts for more than half of the incident cases of HCC in Texas overall [6]. From 1995 to 2010, Hispanics of South Texas had the greatest incidence of HCC (12.1/100,000; 95% CI 11.7–12.5) compared to Hispanics in the rest of Texas (10.9/100,000; 95% 10.6–11.2) and the U.S. (8.4/100,000; 95% CI 8.2–8.7) [13]. The disproportionate incidence of HCC among Hispanics residing in South Texas may be attributed to the region’s distribution of associated risk factors for HCC such diabetes mellitus and obesity, which contribute to NAFLD. Furthermore, there are likely cultural and socioeconomic factors in this population, which serve as barriers to care leading to higher rates of HCC in this region.

## Unique Risk Factors for HCC among Hispanics

Among the general population, there are several well-known risk factors that play an important role in the development of HCC, such as chronic hepatitis B (HBV) and HCV, NAFLD, metabolic syndrome, environmental exposures, and population genetics. It is conceivable that Hispanics of South Texas are disproportionately affected by these risk factors, thus increasing their risk of developing HCC. Although difficult to evaluate precise prevalence of HCV in the U.S., Edlin et al. evaluated the possible underrepresentation of HCV prevalence by analyzing a 2003–2010 U.S. population-based database, National Health and Nutrition Examination Survey (NHANES), and determined at least 1 million persons were excluded and unaccounted for by the database [14]. Despite underreporting of HCV, the overall trend in the prevalence of HCV since 1992 seems to be downtrending from 2.4% to approximately 1.6%, and has remained stable since 2006 [15, 16]. There are limited studies when evaluating for race/ethnicity-specific disparities of HCV, particularly among the Hispanic population. Kuniholm et al. analyzed the NHANES database along with supplementing data from the Hispanic Community Health Study/Study of Latinos (HCHS/SOL) and reported a heterogeneous epidemiological make-up of HCV among Hispanics. Specifically, they found Mexican Americans to have an intermediate HCV prevalence (1.9%; 95% CI 1.1–3.4) compared to low HCV prevalence seen among South American Hispanics (0.4%; 95% CI 0.1–1.9) and high HCV prevalence seen among Puerto Rican Hispanics (11.6%; 95% CI 9.4–14.1) [17]. A randomized, population-based study cohort in a predominantly Mexican American community of south Texas (i.e. Cameron County Cohort) also reported similar findings with a weighted HCV prevalence of 2.3% (*n* = 1,113 95% CI 2.1–3.4%) [18]. Race/ethnicity plays an important role among patients with HCV in the development of HCC. El-Serag et al. demonstrated among 149,407 U.S. Veterans with HCV viremia, only 6% were Hispanic; however, Hispanics had the highest annual incidence rate of cirrhosis (28.8%) and HCC (7.8%) compared to non-Hispanic whites (21.6 and 4.7%, respectively) [19]. Even after adjusting for co-morbid conditions, such as human immunodeficiency virus co-infection, obesity, and diabetes, Hispanics were still at higher risk of developing cirrhosis (hazard ratio (HR) 1.28; 95% CI 1.21–1.37) and HCC (HR 1.61; 95% CI 1.44–1.80) compared to non-Hispanic whites [19].

Due to the growing obesity epidemic, NAFLD has become the leading cause of elevated liver enzymes, where cirrhosis secondary to NAFLD is currently the most rapidly growing indication for liver transplantation within the U.S. [20–22]. Several studies in the past analyzed the NHANES database and determined Hispanics to have the greatest prevalence of NAFLD, ranging between 8.4 and 45%, compared to non-Hispanic whites and African Americans who experienced lower prevalence rates of NAFLD [23–28]. A recent study by Younossi et al. found similar findings when evaluating for race/ethnic disparities among patients with non-alcoholic steatohepatitis (NASH), where being Hispanic was significantly associated with developing NASH (odds ratio (OR) 1.75; 95% CI 1.28–2.33; *p* = 0.0005) [29]. In the setting of NAFLD, studies have shown that obesity and diabetes were independent risk factors for the development of HCC [30, 31]. A meta-analysis of 11 cohort-studies reported that compared to normal weight individuals, overweight patients (body mass index (BMI) 25.1 to 30 kg/m^2^) had a 17% higher risk of developing HCC (relative risk (RR) 1.17; 95% CI 1.02–1.34), whereas obese patients (BMI greater than 30 kg/m^2^) had an increased risk of 89% (RR 1.89; 95% CI 1.51–2.36) [32]. Similarly, Nair et al. evaluated 659 patients with HCC and determined obesity to be an independent predictor for HCC among those with cryptogenic cirrhosis (OR 11.1; 95% CI 1.5–87.4; *p* = 0.02) [33]. It may be difficult to associate obesity with HCC independent of diabetes mellitus, since both disease processes are strongly correlated with one another and are components of metabolic syndrome. Turati et al. conducted a multi-center case–control study to separately explore the association of obesity and diabetes mellitus among patients with ‘hepatitis-free’ HCC in Italy. Among 185 cases of HCC and 404 controls, obese patients were almost twice as likely of developing HCC (OR 1.92; 95% CI 1.03–3.79) and a 4-fold increased likelihood in patients with diabetes mellitus (OR 4.33; 95% CI 1.89–9.86). The risk of HCC further increased when combining more than one component of metabolic syndrome, such as being overweight and having diabetes mellitus (OR 4.75, 95% CI 1.75–12.89) [34]. Similar findings were noted in a U.S. study that calculated population attributable fractions (PAFs), which are utilized to better characterize the impact of specific HCC risk factors by evaluating both exposure and outcome associations along with the prevalence of each exposure in the population. Makarova-Rusher et al. reported metabolic disorders (i.e. obesity, diabetes, metabolic syndrome, and NAFLD) to carry the greatest HCC PAF risk, followed by HCV, alcohol use, smoking, HBV, and genetics disorders. Furthermore, they determined metabolic disorders carried the greatest HCC PAF risk among Hispanics (PAF 39.3%; 95% CI 31.9–46.7%), followed by non-Hispanic whites (PAF 34.8%; 95% CI 33.1–36.5%), Asians (PAF 21.8; 95% CI 16.5–27.1%), and African Americans (PAF 14.4%; 95% CI 6.4–22.3%) [35].

Between 2010 and 2011, approximately two-thirds of adults in the U.S. were at least overweight (68.5%; 95% CI 65.2–71.6%), 35% of those being obese. However, the prevalence of obesity disproportionately affected racial minorities, mainly African Americans (48.1%) and Hispanics (42.5%) [36]. When evaluating the Hispanic population in south Texas (i.e. Cameron County Hispanic Cohort – CCHC), Pan et al. reported 83.1% of their participants being either overweight (32.4%) or obese (50.7%) [37]. Similar trends were observed with diabetes mellitus where the overall prevalence of diabetes in the U.S. is estimated to be approximately 9.3%; however, the CCHC had a prevalence of 27.6% [37, 38]. The high prevalence of obesity and diabetes in Cameron County are likely contributing to the high rates of NAFLD [39, 40], which may explain why the annual incidence of HCC in this unique population is greater than Hispanics in other parts of the U.S.

Although obesity and insulin resistance are major contributing factors for the development of hepatic steatosis, the heterogeneous prevalence of hepatic steatosis between race/ethnicities may suggest other influencing factors, such as genetic variance [41]. From the Dallas Heart Study, Romeo et al. first demonstrated that the rs738409 [G] allele in the patatin-like phospholipase domain-containing 3 (*PNPLA3*) gene was significantly associated with increased hepatic fat levels (*p* = 5.9 × 10^−10^) and hepatic inflammation (*p* = 3.7 × 10^−4^), where the allele was more commonly seen among Hispanics (0.49) compared to non-Hispanic whites (0.23) and African Americans (0.17) [42]. Similarly, Wagenknecht et al. demonstrated *PNPLA3* rs738409 [G] to be two times more common in Hispanics compared African Americans (40% vs. 19%), which seems consistent with the greater prevalence of NAFLD among Hispanics compared to African Americans (24% vs. 9%) [43]. In addition to race/ethnic variance, G allele homozygotes have greater than a twofold increase of hepatic fat content compared to noncarriers [42]. *PNPLA3* genetic polymorphism may influence not only hepatic fat accumulation but also contribute to a more aggressive disease course. A meta-analysis of 16 studies performed by Sookoian et al. concluded GG homozygotes to have not only 73% higher hepatic fat content, but also a 3.24-fold greater risk for necroinflammatory scores and 3.2-fold greater risk of developing fibrosis compared to the CC homozygote control (*p* < 1.0 × 10^−9^). Furthermore, GG homozygotes were significantly more likely to develop NASH compared to the control group (OR 3.488; 95% CI 1.859–6.545; *p* < 2.0 × 10^−4^) [44].

## Factors causing disparities in South Texas

As alluded to earlier, Asians have the highest incidence of overall HCC in the U.S. followed by African Americans, Hispanics, and Non-Hispanic whites; however, recent trends have noted the greatest percent increase in HCC incidence among Hispanics whereas Asians are experiencing a decline in the incidence of HCC [4, 45]. Furthermore, Hispanic individuals have a 5-fold greater risk of HCC mortality based on the Third NHANES, a nationwide survey from 1988 to 1994, with Hispanic ethnicity being an independent predictor of mortality (HR 5.14; 95% CI 1.75–15.06) [46]. Although the disease process of HCC and its associated risk factors, which include but are not limited to diabetes, NAFLD, HCV, HBV and metabolic syndrome, play an important role in race/ethnicity specific disparities of HCC, it is also important to understand complex psychosocial influences which may contribute to disparities seen in healthcare where it affects ethnic minorities to a greater degree.

Alcohol use, either as a primary factor or in combination with HBV, HCV or diabetes, is a key culprit for the development of HCC. The effect of alcohol on the liver is well studied where chronic alcohol use greater than 80 grams per day for more than 10 years leads to a 5-fold increase in the risk of HCC [47]. We want to focus on race/ethnic disparities in alcohol consumption, specifically affecting the Hispanic population since it is the largest and fastest growing minority group in the U.S. According to the 2012–2013 longitudinal study from the National Epidemiologic Survey on Alcohol and Related Conditions (NESARC), Hispanics had the greatest prevalence of heavy drinking (31.6%) compared to other race/ethnic minorities [48]. Additionally, cross-sectional and longitudinal data noted a declining trend of alcohol consumption in the U.S. when people reach their 30s; however, alcohol consumption in Blacks and Hispanics decreases less dramatically with age [48, 49]. One study analyzed the health of Hispanic men, including drinking habits, living on the Texas-Mexico border in an area with a 93.2% Hispanic population. This cross-sectional study included 945 Hispanic men in the CCHC from Brownsville, Texas between 2004 through 2015. In the study, “heavy drinking” was defined as more than 4 drinks on any day or 14 drinks per week and found that 7.3% reported heavy drinking and 60.3% reported occasional alcohol consumption [50].

Several theories have been proposed to explain the differences in drinking patterns among various racial/ethnic groups, including degree of acculturation and level of awareness of racial/ethnic stigma. Acculturation refers to how immigrants adapt to the behaviors, values and culture of the host society. One proposed theory is that more acculturated Hispanics have higher levels of alcohol use since acculturation has been associated with more liberal views towards drinking [51]. The Hispanic Americans Baseline Alcohol Survey (HABLAS) was used to study alcohol patterns in 5 major metropolitan areas in the United States. They concluded that Hispanic men of Puerto Rican and Mexican origin consume a higher number of drinks on average compared with Hispanics of Cuban or Dominican origin. This study also found that regardless of country of origin, the number of drinks per week for women, for instance, was positively correlated with level of acculturation [49]. In a prospective study of 36,864 Hispanics (18,485 US-born and 18,379 foreign-born) from Hawaii and California, the incidence of HCC was statistically greater in US-born Hispanics compared with foreign-born Hispanics even when controlling for age and gender (HR 1.61; 95% CI 1.20–2.17) [52]. It is conceivable that these observations are attributed to greater prevalence of HCC risk factors (e.g. HCV, alcohol consumption, and metabolic syndrome) among more acculturated US-born Hispanics [53]. Although there is no specific data on the population distribution of US-born versus foreign-born Hispanics in South Texas, U.S.-born Hispanics constitute 70% of the Hispanic population in Texas whereas foreign-born Hispanics account for 30% of Hispanics in Texas [54]. Another theory compared the level of awareness of racial/ethnic stigma on drinking patterns. A 2005 U.S. National Alcohol Survey, a nationally representative telephone-based survey of adults ages 18 and older, showed that Hispanics who self-reported experiences of racial/ethnic discrimination had a 2-fold greater risk of problem drinking [52]. Similarly, another study showed that Hispanics who self-reported major or lifetime discrimination attributed to race or ethnicity were 62% more likely of drinking heavily (OR 1.62; 95% CI 1.10–2.40) [55]. To our knowledge, there have not been studies specifically looking at the degree of racial/ethnic discrimination experienced by South Texas Hispanics. As mentioned previously, Hispanics of Mexican origin are the predominant Hispanic group in South Texas and studies have shown that Mexican Americans who are darker, more educated, and have more contact with Whites experience more discrimination [56].

Access to healthcare plays a vital role in treatment and survival in patients with HCC. A retrospective cohort study using SEER HCC registry evaluated early invasive therapy (i.e. tumor ablation, hepatic resection, liver transplant) and analyzed variations in survival across racial/ethnic groups. The study demonstrated median survival improvement from 4 to 6 months without treatment to 40 months with treatment among Hispanics. However, decreased utilization of invasive treatment strategies were noted among African American and Hispanic patients with HCC. In turn, there were higher rates of mortality among African Americans (HR 1.24; 95% CI 1.15–1.33) and Hispanic patients (HR 1.08; 95% CI 1.01–1.15) and lower rates of mortality in Asians (HR 0.87; 95% CI 0.82–0.93) compared with non-Hispanic whites [57]. Similarly, Altekruse et al. characterized trends of HCC mortality from 2000 to 2010 and noted significantly increased rates of HCC mortality among non-Hispanic whites, African Americans, and Hispanics, whereas decreased rates of HCC mortality was seen among Asians [9]. Although the exact etiology behind these disparate findings is unclear, these trends may be from greater awareness of HBV and greater awareness and implementation of HCC screening and surveillance programs among patients with chronic HBV, many of whom are Asian.

## Conclusion

In summary, race/ethnicity-specific disparities persist in the epidemiology of HCC. Hispanics, particularly populations residing in south Texas, seem to experience more disparate trends in the incidence and prevalence of HCC. These may be attributed to increased exposures to well known risk factors, such as excessive alcohol consumption, obesity and diabetes mellitus, in turn resulting in the development of NAFLD/NASH. In addition, population genetics may predispose this unique race/ethnic cohort to a greater propensity of developing HCC compared to other race groups. Current literature suggests complex psychosocial factors that further complicate disparities seen in HCC including degree of acculturation, access to healthcare, and provider-specific variables of HCC screening and surveillance of at-risk patients. Improved awareness in screening and surveillance modalities of HCC among Hispanics are important in guiding future research for targeted preventive and therapeutic interventions.

## Abbreviations

BMI: Body mass index
CCHC: Cameron county Hispanic cohort
HBV: Hepatitis B virus
HCC: Hepatocellular carcinoma
HCV: Hepatitis C virus
NAFLD: Non-alcoholic fatty liver disease
NASH: Non-alcohoic steatohepatitis
NESARC: National epidemiologic survey on alcohol and related conditions
NHANES: National health and nutritional examination survey
SEER: Surveillance, epidemiology, and end results
U.S.: United States
